# Interpregnancy interval and early infant neurodevelopment: the role of maternal–fetal glucose metabolism

**DOI:** 10.1186/s12916-023-03191-0

**Published:** 2024-01-02

**Authors:** Ruirui Ma, Peng Wang, Qiaolan Yang, Yuanyuan Zhu, Lei Zhang, Yuhong Wang, Lijun Sun, Wenxiang Li, Jinfang Ge, Peng Zhu

**Affiliations:** 1 Department of Maternal, Child and Adolescent Health, School of Public Health, Anhui Medical University, 81 Meishan Road, Hefei, 230032 Anhui China; 2 MOE Key Laboratory of Population Health Across Life Cycle, Anhui Medical University, Hefei, China; 3 NHC Key Laboratory of Study On Abnormal Gametes and Reproductive Tract, Anhui Medical University, Hefei, China; 4 Anhui Provincial Key Laboratory of Population Health and Aristogenics, Anhui Medical University, Hefei, China; 5 Center for Big Data and Population Health of IHM, Anhui Medical University, Hefei, China; 6 Department of Urology, The First Affiliated Hospital of Anhui Medical University, Hefei, China; 7 School of Pharmacy, Anhui Medical University, 81 Meishan Road, Hefei, 230032 Anhui China

**Keywords:** Interpregnancy interval, Neurodevelopment, Glucose, HOMA-IR

## Abstract

**Background:**

Interpregnancy interval (IPI) is associated with a variety of adverse maternal and infant outcomes. However, reports of its associations with early infant neurodevelopment are limited and the mechanisms of this association have not been elucidated. Maternal–fetal glucose metabolism has been shown to be associated with infant neurodevelopmental. The objective of this study was to determine whether this metabolism plays a role in the relationship between IPI and neurodevelopment.

**Methods:**

This prospective birth cohort study included 2599 mother-infant pairs. The IPI was calculated by subtracting the gestational age of the current pregnancy from the interval at the end of the previous pregnancy. Neurodevelopmental outcomes at 12 months in infants were assessed by the Ages and Stages Questionnaire Edition 3 (ASQ-3). Maternal fasting venous blood was collected at 24–28 weeks and cord blood was collected at delivery. The association between IPI and neurodevelopment was determined by logistic regression. Mediation and sensitivity analyses were also conducted.

**Results:**

In our cohort, 14.0% had an IPI < 12 months. IPI < 12 months increased the failure of the communication domain, fine motor domain, and personal social domain of the ASQ (relative risks (RRs) with 95% confidence interval (CI): 1.73 [1.11,2.70]; 1.73 [1.10,2.72]; 1.51 [1.00,2.29]). Maternal homeostasis model assessment of insulin resistance (HOMA-IR) and cord blood C-peptide was significantly associated with failure in the communication domain [RRs with 95% CI: 1.15 (1.02, 1.31); 2.15 (1.26, 3.67)]. The proportion of the association between IPI and failure of the communication domain risk mediated by maternal HOMA-IR and cord blood C-peptide was 14.4%.

**Conclusions:**

IPI < 12 months was associated with failing the communication domain in infants. Maternal–fetal glucose metabolism abnormality may partially explain the risk of neurodevelopmental delay caused by short IPI.

**Supplementary Information:**

The online version contains supplementary material available at 10.1186/s12916-023-03191-0.

## Background

Interpregnancy interval (IPI, the length of time between pregnancies) is a potentially modifiable risk factor that has been reported to have an effect on pregnancy and child development [1, 2]. In China, the proportion of births with a short IPI is increasing, owing to the more frequent delay in childbearing age and the introduction of a three-child family planning policy [3, 4]. A short IPI has been reported to increase the risk of mental illness in offspring [2, 5]. Similar studies have been conducted in patients with schizophrenia [6]. However, most previous studies have concentrated on Autism spectrum disorder (ASD) and did not extend their analysis to other general neurodevelopmental disorders. Neurodevelopmental delays are thought to be caused by a complex combination of factors. These factors include genetics, the environment, prenatal and perinatal conditions, and the postnatal environment [7]. In less than half of children, with developmental delays, these were detected before school entry, with the vast majority of children not receiving any intervention in the early pre-school years [8]. Therefore, an improved early screening for neurodevelopmental delays will provide useful interventions and support before admission to school. The Ages and Stages Questionnaire (ASQ) is an age-specific, structured, parent-reported developmental screening test for children aged between 1 and 66 months of age [8]. The ASQ-3 has been validated in many countries due to its ease of administration, short completion time, easy interpretation, and the ability to dramatically improve the clinical identification of children with suspected developmental delays [9, 10]. However, few studies have examined the relationship between a short IPI and early developmental delay.

Currently, the mechanisms by which a short IPI affects neurodevelopment in infants remain unclear. Nutrient depletion, persistent maternal inflammation, increased maternal psychological stress, gestational diabetes, and obesity are more likely to occur after a short IPI [2, 11]. Clinical studies have shown that these maternal metabolic conditions are associated with insulin resistance, high levels of C-peptides, and altered glucose metabolism in infants [12, 13]. Insulin resistance and cerebral glucose metabolism have received considerable attention as factors involved in the pathogenesis of cognitive impairment. Insulin crosses the blood–brain barrier and interacts with receptors located in brain regions of the brain involved in the regulation of energy balance and glucose metabolism, as well as in the modulation of learning and memory [14]. Maternal insulin resistance causes fetal hyperinsulinemia (cord-blood serum C-peptide levels above the 90th percentile) and hypoglycemia [15]. Epidemiological and animal studies have shown that fetal hyperinsulinemia and hypoglycemia are significantly associated with an increased risk of neurodevelopmental disorders such as ASD [16, 17]. Therefore, we hypothesized that maternal–fetal glucose metabolism disorders may be linked with short IPI and neurodevelopment in infants.

In this prospective birth cohort study, our objective was to investigate the association between IPI and early neurodevelopment in infants and to extend previous studies by controlling for additional potential confounders. Furthermore, we examined the hypothesis that maternal–fetal glucose metabolism disorders mediate the association between a short IPI and neurodevelopmental.

## Methods

### Study design and participants

We recruited 5731 pregnant women from Anhui Women and Child Health Care Hospital, the First People’s Hospital of Hefei City, and the First Affiliated Hospital of Anhui Medical University, aged 18 to 45 years, with gestational ages between 16 to 23 weeks, living in Hefei (situated in the eastern part of China, in the Anhui Province), speaking Chinese, and with pregnancy experience. This was a population-based, prospective birth cohort study, and survey process for this cohort has been previously described in detail [18]. This study aimed to evaluate the relationship between metabolism during pregnancy and the growth and neurodevelopment in infants from March 2018 to July 2022. The study’s exclusion criteria included communication difficulties, previous pregnancy ending in stillbirth, previous birth defect, abnormal liver, kidney, or thyroid function, and missing blood samples. Finally, we collected complete data from 5128 pregnant women.

The study conducted four main surveys, with the first administered at 16–23 weeks of gestation. Participants completed a structured questionnaire covering sociodemographic characteristics, perinatal health status, diet, and lifestyle through face-to-face interviews. The second survey was administered at 24–28 weeks of gestation, when a specialized nurse collected fasting venous blood samples from pregnant women and obtained blood glucose and insulin levels from the electronic medical system. The third survey was administered at the time of delivery, when the basic characteristics of the newborn (sex, gestational age, date of birth, weight, and mode of delivery) were obtained from electronic medical records, and cord blood was collected by a professional nurse. The final survey was administered at the follow-up of the mother, when the child was 12 months old. Finally, the ASQ-3 at 12 months postpartum was obtained for 2599 mother-infant pairs. Details of the recruitment of pregnant women are shown in Fig. S1.

### Determination of interpregnancy interval

The IPI was calculated by subtracting the gestational age of the current pregnancy from the interval at the end of the previous pregnancy. Currently, the definition of a short IPI is inconsistent in the available literature. It has been defined in different studies as < 6 to 36 months of age. However, two studies found that an IPI < 12 months was associated with the highest risk of poor neurodevelopment [19, 20], therefore, the present study focused on the results of studies with an IPI of less than 12 months.

### Measurement of HOMA-IR and cord blood markers

Fasting venous blood was drawn from each pregnant woman at 24–28 weeks of gestation to perform a standard 75 g oral glucose tolerance test (OGTT), and plasma glucose levels were measured at 0, 60, and 120 min. Fasting blood glucose and insulin levels of pregnant women were obtained from the hospital's electronic medical record system. Homeostasis model assessment of insulin resistance (HOMA-IR) was calculated using the equation HOMA-IR = fasting insulin (µU/mL) × fasting plasma glucose (mmol/L)/22.5 [21]. Cord blood was collected at delivery to detect metabolic markers, including C-peptide levels, which were measured using an electrochemical luminescence detection kit (Roche E601; Sandhofer, Mannheim, Germany), according to the manufacturer’s instructions. The intra- and inter-subject coefficients of variation were < 10%.

### Neurodevelopment screening

The ASQ-3 is a vital instrument for screening infants for the risk of early developmental delays. The ASQ-3 surveys five developmental skill domains: communication, gross motor, fine motor, problem-solving, and personal-social skills [10]. The parent or caregiver completed the ASQ-3 questionnaire screening when the infant was 12 months old. Each dimension had six questions, with possible responses: “yes” (ten points) if the child performed the behavior, “sometimes” (5 points) indicated that the skill was emerging, and “not yet” (0 points) indicated that the child was not yet able to perform the specific activity. Six problem scores were added for each dimension, and each dimension’s score reflected the child's ability in that dimension. Abnormal ASQ-3 outcomes were described as 2 standard deviations (SDs) below the mean score [13].

We generated a broader outcome variable based on the number of areas in which the child demonstrated risk of neurodevelopmental delay, using data from the ASQ-3 to boost statistical power, which was defined as follows: (1) delay in communication development (score ≤ 2 SD for age on the ASQ-3 communication domain); (2) delay in motor development (score ≤ 2 SD for age on the ASQ-3 fine motor domain), and (3) delay in social development (score ≤ 2 SD for age on the ASQ-3 personal-social domain). Hence, this variable the captured a delay in all 3 domains (scored as 3), with a delay in any 2 (scored as 2), a delay in any 1 (scored as 1), and no delay in any area (scored as 0) [22].

### Potential covariates

The covariates included perinatal parental factors and infant characteristics. Perinatal parental factors included maternal age (≥ 30 or < 30 years), education level (≥ 12 or < 12 years of completed schooling), income (≥ 4000 or < 4000 RMB/month) [23] (at March 2018, the exchange rate for 4000 RMB was approximately 635 USD), parity (< 3 or ≥ 3 children), prepregnancy overweight/obesity (body mass index [BMI] ≥ or BMI < 24; BMI was calculated as weight (kg)/height (m)^2^], folic acid supplementation (yes or no), anemia during pregnancy (yes or no), systolic blood pressure (SBP, mmHg); diastolic blood pressure (DBP, mmHg), gestational diabetes mellitus (yes or no), depression during pregnancy (yes or no), and the frequency of moderate physical exercise in the past three months prior were evaluated using the International Physical Activity Questionnaire (< 3 and ≥ 3 days/week of not less than 10 min per day) [24]. Infant factors include sex (male and female), mode of delivery (vaginal delivery or cesarean section), birth weight (g), and gestational week at birth (≥ 37 or < 37 weeks).

Furthermore, we further controlled for postnatal factors that may affect neurodevelopment in infants [7], which were obtained through follow-up questionnaires at 6 and 12 months. These included postpartum depression (yes or no), the number of siblings (< 2 or ≥ 2), the duration of exclusive breastfeeding (< 6 or > 6 months), feeding difficulties (yes or no), poor sleeping in infants (yes or no), fever in infants ≥ 38.5 °C (yes or no), and parents as the primary caregivers (yes or no). The parental feeding questionnaire at 6 months consisted of 23 items, of which 21 items had response options of “Never,” “Occasionally,” “Sometimes,” or “Often,” with scores of 4, 3, 2, and 1 respectively), with higher scores indicating more frequent behavior; two items had response options of “yes/no” (for the evaluation of feeding in children aged 2 to 3 years). A score of > 34 (> P_75_) on the questionnaire was considered indicating feeding difficulty [25]. The criteria used to define poor sleepers on the basis of the Brief Infant Sleep Questionnaire (BISQ) at 6 months measures were as follows: (1) the child wakes > 3 times per night; (2) nocturnal wakefulness is > 1 h; or (3) the total sleep time is < 9 h [26].

Confounders related to IPI and infants’ risk of neurodevelopmental delays were determined using a directed acyclic graph (DAG) to visualize these relationships (Fig. S2).

### Statistical analysis

Demographic characteristics and clinical information were compared between the different IPI groups, using Student’s *t* test for continuous variables and classified variables using *χ*^2^ tests. Multiple logistic regression models were used to estimate the relationship between the IPI and neurodevelopmental outcomes. Multiple confounder variables were adjusted for in different models. Model 1 was adjusted for sociodemographic characteristics. Model 2 was adjusted for perinatal health status and lifestyle. Model 3 was adjusted for the birth outcome. Model 4 was adjusted for postnatal factors. The relationship between HOMA-IR and C-peptide levels was assessed by linear regression analysis. The clinical diagnosis of fetal hyperinsulinemia was used to create a dichotomous variable of cord blood serum C-peptide levels above the 90th percentile (P_90_). The relationships between HOMA-IR, C-peptide levels above P_90_, and ASQ-3 domains were evaluated using regression analysis. All regression models were adjusted for the variables listed in the covariates section. Results are expressed as relative risk (RR) and 95% confidence interval (CI).

When the outcome of interest was rare (usually defined as an incidence < 10%), RR approximated the odds ratio (OR)/(1 − P_0_ + P_0_ × OR) [27], where P_0_ represented the incidence or probability of an outcome in the unexposed group. We used this formula to correct the adjusted OR obtained from logistic regression analysis and derived an estimate of an association or treatment effect that better represented the true RR.

A mediation analysis was performed using the SPSS PROCESS plug-in. Before testing the mediation, we ensured that the criteria for mediation criteria were met, namely that the predictor, mediator, and outcome variables were interrelated. We evaluated the role of maternal HOMA-IR and cord blood C-peptide in the association between the IPI and the failure of the communication domain. For ease of interpretation, we normalized the levels of HOMA-IR and cord blood C-level peptides using a natural logarithm transformation. Decomposing the total effects into direct and indirect paths calculated the proportion of total effects mediated by the indirect path. These analyses allowed quantification of the total effect (the association between IPI and failure in the communication domain), a direct effect (the total effect without the influence of mediators), and an indirect effect (the effect of IPI on failure in the communication domain attributable to mediators). The effect sizes for the mediation analysis are shown as RR and 95% CI*.*

Four sensitivity analyses were also performed to test the robustness of the findings: (1) we progressively adjusted the confounders from Model 1 to Model 4 based on the DAG; (2) we analyzed the association of continuous variables IPIs and more finely categorized IPIs with the risk of neurodevelopmental delay; (3) we used Poisson regression analysis to validate the association between IPI and the risk of neurodevelopmental delay; and (4) we used the correlation residual method to examine whether the observed mediated effect was robust to potential confounding by some unmeasured variables. Imai et al. proposed a sensitivity analysis for causal mediation based on the correlation between the error for the mediation model and the error for the outcome model [28]. They denoted this correlation across the two error terms as ρ, which served as the sensitivity parameter. Such a correlation can arise if there are omitted variables that affect both the mediator and outcome variables, because these omitted variables will be part of the two error terms. Thus, under sequential ignorability, equals zero, and non-zero values of ρ imply a departure from the ignorability assumption. Imai et al. showed that it is possible to express the average causal mediation effect as a function of ρ and model parameters that can be consistently estimated even though ρ was non-zero. The total effects included the average mediation effect (AME) and the average direct effect (ADE).

All analyses were performed using SPSS version 26.0 software (IBM Corp.) and in R (version 3.5.0; R Core Development Team) using the R statistical packages “ggplot2,” “car,” and “mediation.” All *P*-values were 2-tailed, and statistical significance was established at a *P* < 0.05.

## Results

Characteristics of the study population are summarized in Table 1. Of the 5128 enrolled participants, 717 (14.0%) had an IPI of < 12 months. Table 1 shows that the short IPI maternal age of IPI was younger (29.27 ± 4.1 vs. 31.10 ± 3.9, *P* < 0.001), had a higher DBP (69.58 ± 7.6 vs. 68.86 ± 7.5, *P* = 0.017) and longer gestational weeks of delivery (39.03 ± 1.2 vs. 38.87 ± 1.4, *P* = 0.005). The incidence of shorter IPI, income < 4000 RMB/month (76.1% vs. 69.3%, *P* < 0.001), depression during pregnancy (16.5% vs. 13.1%, *P* = 0.015), and postpartum depression (36.9% vs. 26.4%, *P* = 0.008) was higher, whereas the incidence of cesarean delivery (33.4% vs. 37.8%, *P* = 0.042) was lower. Infants born to mothers with an IPI < 12 months were more inclined to fail in the communication, fine motor, and personal-social domains than those with a longer IPI (8.8% vs. 4.6%, *P* = 0.001; 8.0% vs. 4.7%, *P* = 0.009; 9.9% vs. 6.2%, *P* = 0.008). Regarding other sociodemographic characteristics, characteristics associated with short IPIs were largely similar to those of long IPIs.

**Table 1:** Characteristics of the sample (mean ± SD or *n* (%))

	Interpregnancy interval, months		*P* value
	< 12 (*n* = 717)	≥ 12 (*n* = 4411)	
**Sociodemographic characteristics**			
Maternal age, years	29.27 ± 4.1	31.10 ± 3.9	** < 0.001**
Maternal education < 12 years	332 (46.3%)	2037 (46.2%)	0.971
Income < 4000 RMB/month	543 (75.7%)	3029 (68.7%)	** < 0.001**
**Perinatal health status and lifestyle**			
Parity ≥ 3	376 (52.4%)	1939(44.0%)	** < 0.001**
Prepregnancy overweight/obesity	170 (23.7%)	959(21.7%)	0.238
SBP, mmHg	110.90 ± 10.2	110.19 ± 10.0	0.081
DBP, mmHg	69.58 ± 7.6	68.86 ± 7.5	**0.017**
Depression during pregnancy	118 (16.5%)	575 (13.0%)	**0.015**
Gestational diabetes mellitus	130 (18.1%)	907 (20.6%)	0.124
Anemia during pregnancy	252 (35.1%)	1703 (38.6%)	0.117
FPG, mmol/L	4.54 (4.27–4.84)	4.54 (4.28–4.86)	0.796
Insulin, IU/L	8.15 (6.10–10.98)	7.80 (5.7–10.4)	0.051
Vitamin D supplement	358(49.9%)	2384 (54.0%)	0.172
Folic acid supplement	309(43.1%)	1843 (41.8%)	0.567
Physical activity < 3 days/week	388(54.1%)	2478 (56.2%)	0.248
**Birth outcomes**			
Cesarean section	239(33.3%)	1667 (37.8%)	**0.042**
Male	397(55.4%)	2337 (53.0%)	0.500
Gestational week at birth, week	39.03 ± 1.2	38.87 ± 1.4	**0.005**
Birth weight, g	3458 ± 450	3431 ± 450	0.179
**Postnatal factors**			
Postpartum depression	264 (36.8%)	1164 (26.4%)	**0.008**
Exclusive breastfeeding > 6 months	574 (80.1%)	3564 (80.8%)	0.849
Feeding difficulties	154 (21.5%)	782 (17.7%)	0.260
Poor sleeping in infants	158 (22.0%)	930 (21.1%)	0.768
Fever in infants ≥ 38.5 °C	384 (53.6%)	2311 (52.4%)	0.890
Parents as primary caregivers	472 (65.8%)	3013 (68.3%)	0.535
Number of siblings ≥ 2	286 (39.9%)	1653 (37.5%)	0.762
**ASQ-3 at 12 months**^a^			
Scores for communication domain	52.6 ± 10.1	54.3 ± 8.2	**0.032**
Failure to communication domain	32 (8.8%)	104 (4.6%)	**0.001**
Scores for gross motor domain	46.4 ± 13.9	46.1 ± 13.7	0.807
Failure to gross motor domain	17(4.7%)	102(4.6%)	0.908
Scores for fine motor domain	51.7 ± 11.3	53.1 ± 9.6	0.058
Failure to fine motor domain	29 (8.0%)	106 (4.7%)	**0.009**
Scores for problem-solving domain	50.8 ± 10.6	50.9 ± 10.4	0.922
Failure to problem-solving domain	12(3.3%)	67(3.0%)	0.742
Scores for personal social domain	47.6 ± 12.4	49.3 ± 11.5	**0.010**
Failure to personal social domain	36 (9.9%)	138 (6.2%)	**0.008**

At the March 2018 exchange rate, 4000 RMB ≈ 635 USD

*SD* standard deviation, *RMB* Chinese yuan, *SBP* systolic blood pressure, *DBP* diastolic blood pressure, *FPG* fasting plasma glucose, *ASQ-3* The Ages and Stages Questionnaires Edition 3

^a^Total number of children is 2599

The association of confounders with the risk of neurodevelopmental delay and maternal–fetal glucose metabolism indicators is shown in Tables S1 and S2. We compared the baseline characteristics of the study participants and of nonparticipants, and, as shown in Table S3, the differences in the baseline characteristics were not statistically significant.

### IPI and ASQ failure of offspring

This study estimated the relationship between the IPI and dichotomous infant neurodevelopmental outcomes and between the IPI and the number of delays in the 3 domains (Table 2). In the adjusted analysis, an IPI shorter than 12 months was associated with an increased risk of failure of the communication domain [RR with 95% CI: 1.73 (1.11,2.70)]. An IPI < 12 months was also associated with an increased risk of failure in the fine motor and personal-social domains (RRs with 95% CI: 1.73 [1.10,2.72] and 1.51 [1.00,2.29], respectively).

**Table 2:** Associations of the interpregnancy interval with infant neurodevelopment

ASQ at 12 months	*N* (%)	Interpregnancy interval [*RR* (95% *CI*)]			
		Model 1	Model 2	Model 3	Model 4
**Delay in one ASQ domain**					
Communication	136 (5.2%)	**1.99 (1.32,3.01)**	**1.80 (1.18,2.75)**	**1.78 (1.16,2.74)**	**1.73 (1.11,2.70)**
Gross motor	119 (4.6%)	1.03 (0.61,1.75)	1.12 (0.66,1.91)	1.08 (0.63,1.85)	1.08 (0.62,1.88)
Fine motor	135 (5.2%)	**1.75 (1.14,2.68)**	**1.74 (1.13,2.69)**	**1.76 (1.13,2.72)**	**1.73 (1.10,2.72)**
Problem-solving	79 (3.0%)	1.11 (0.60,2.07)	1.11 (0.58,2.14)	1.04 (0.54,2.02)	1.08 (0.56,2.11)
Personal social	174 (6.7%)	**1.68 (1.14,2.47)**	**1.61 (1.09,2.39)**	**1.59 (1.06,2.36)**	**1.51 (1.00,2.29)**
**Delay in communication, fine motor, and personal social domain**					
Delay in any 1 domain	335 (12.9%)	**1.63 (1.21,2.19)**	**1.57 (1.16,2.13)**	**1.57 (1.15,2.13)**	**1.50 (1.09,2.06)**
Delay in any 2 domains^a^	67 (2.6%)	**2.53 (1.47,4.35)**	**2.20 (1.25,3.86)**	**2.17 (1.22,3.83)**	**2.12 (1.17,3.83)**
Delay in all 3 domains^b^	42 (1.6%)	**2.84 (1.46,5.51)**	**2.69 (1.37,5.30)**	**2.55 (1.28,5.08)**	**2.38 (1.17,4.88)**
**Number of delay across 3 domains**^c^	-	**2.02 (1.36,2.99)**	**1.96 (1.30,2.97)**	**1.94 (1.28,2.95)**	**1.87 (1.22,2.88)**

Model 1 was adjusted for sociodemographic characteristics including maternal age, education, income

Model 2 was further adjusted for perinatal health status and lifestyle including parity, prepregnancy overweight/obesity, SBP, DBP, depression during pregnancy, gestational diabetes mellitus, anemia during pregnancy, vitamin D supplement, folic acid supplement, and physical activity

Model 3 was further adjusted for the birth outcomes including delivery mode, infant gender, gestational week, and birth weight

Model 4 was further adjusted for postnatal factors including postpartum depression, number of siblings, duration of exclusive breastfeeding, feeding difficulties, poor sleeping in infants, high fever in infants, and primary caregiver

^a^ Poisson regression analysis revealed that the *IRR* with 95% *CI* between IPI and delay in any 2 domains was 1.11 (1.01,1.23)

^b^ Poisson regression analysis revealed that the *IRR* with 95% *CI* between IPI and delay in all 3 domains was 1.13 (1.01,1.25)

^c^ For the number of domains of delay across communication, fine motor, and personal social domain, 0 = no delay in any domain, 1 = 1 delay in any domain, 2 = 2 delays in any domain, and 3 = delay in all 3 domains

After adjustment for confounding effects, the RR with 95% CI between IPI and delay in any 1 domain of the communication, fine motor, and problem-solving domains was 1.50 (1.09,2.06), delay in any 2 domains was 2.12 (1.17,3.83), and delay in all 3 domains was 2.38 (1.17,4.88). Further analysis found that a shorter IPI was associated with a higher RR of a higher number of child risk of neurodevelopmental delay areas, with a correlation of 1.87 (1.22,2.88).

### HOMA-IR and cord blood C-peptide with ASQ failure

**Fig. 1:**
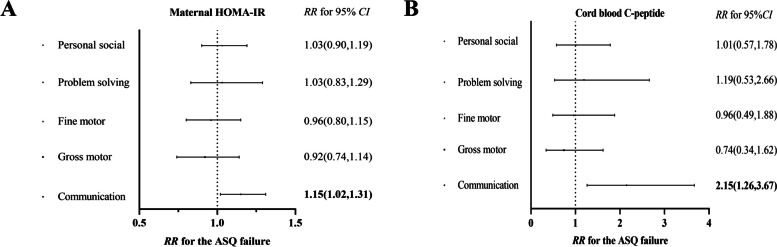
Associations of maternal HOMA-IR and cord blood C-peptide with risks of ASQ failure. **A** RRs with 95% CI for the dichotomous outcomes of ASQ failures including communication domains, gross motor domains, fine motor domains, problem-solving domains, and personal social domains, are for cord blood C-peptide levels above the 90th percentile. **B** RRs with 95% CI for the dichotomous outcomes of ASQ failures including communication domains, gross motor domains, fine motor domains, problem-solving domains, and personal social domains, are for maternal HOMA-IR level

Maternal HOMA-IR and cord blood C-peptide were higher following a short IPI (Fig. S3A, B). Moreover, maternal HOMA-IR and cord blood C-peptide levels showed a weaker association in mothers with a longer IPI, the β-values with 95% CI for the short and long IPIs were 0.080 (0.041,0.118) and 0.055 (0.026,0.084), respectively (Fig. S3C). There was a positive association between maternal HOMA-IR and infant failure in the communication domain (RR with 95% CI: 1.15 [1.02,1.31]) (Fig. 1A). The cord blood C-peptide analysis showed a positive relationship between cord blood serum C-peptide levels above the 90th percentile and failure of the communication domain in infants (RR with 95% CI: 2.15 [1.26,3.67]) (Fig. 1B).

**Fig. 2:**
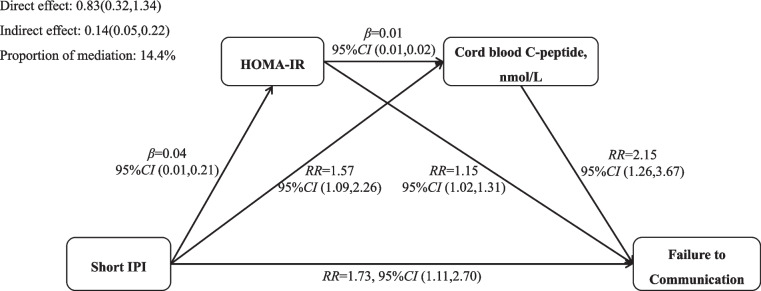
Mediation effects of maternal HOMA-IR (log-transformed) and cord C-peptide (log-transformed) on the relationship between short IPI and the risks of failing the communication domain. The total indirect effect consists of 3 pathways. Pathway 1 (Short IPI → HOMA-IR → Failure to communication): 2.1%; Pathway 2 (Short IPI → Cord blood C-peptide → Failure to communication): 11.3%; Pathway 3 (Short IPI → HOMA-IR → Cord blood C-peptide → Failure to communication): 0.5%

The role of maternal HOMA-IR and cord blood C-peptide in the association between a short IPI and infant failure in the communication domain was evaluated using a mediation analysis (Fig. 2). The mediation analysis showed a direct contribution of a short IPI to the failure of the communication domain failure (excluding mediated effects) (direct effect with 95% CI: 0.83 (0.32,1.34)]. Maternal HOMA-IR and log-transformed cord blood C-peptides values may play a mediating role in the association of short IPI with failure in the communication domain (indirect effect with 95% CI: 0.14 (0.05,0.22)] and indirect effects accounted for 14.4% of the total effect.

### Sensitivity analysis

An IPI < 12 months was associated with a risk of neurodevelopmental delay, and ≥ 12 months was not statistically associated with a risk of neurodevelopmental delay (Fig. S4). A higher risk of neurodevelopmental delay was similarly found to be associated with IPIs < 3, 3–5, and 6–11 months in the more refined IPI subgroups (Tables S4 and S5), despite the wider CIs. We also found that the levels of HOMA-IR and C-peptides from cord blood C-peptide levels were higher in patients with IPIs < 3, 3–5, and 6–11 months (Fig. S5). Considering that the incidence of delays in any 2 domains and delays in all 3 domains was less than 5%, we added a sensitivity analysis with a Poisson regression analysis and found these significant associations. Our results showed that the incident rate ratio (IRR) with 95% CI between IPI and delays in any 2 domains was 1.11 (1.01,1.23), and the delay in all 3 domains was 1.13 (1.01,1.25). We performed a sensitivity analysis based on the coefficient of determination (Fig. S6). The left plot *Y*-axis indicates the average mediation effect value and the *X*-axis indicates the sensitivity parameter *ρ*. The dashed line indicates when *ρ* = 0, that is, when there is no confounding effect, and the solid line indicates the value of ρ when the confounding effect leads to the disappearance of the mediation effect, and the shaded part is the confidence interval. The higher the absolute value of the *ρ* indicates a stronger confounding effect, and, the corresponding result of the mediation effect is more reliable. In Fig. S6, the right plot presents the sensitivity analysis based on the coefficients of determination, ${\widetilde{R}}_{M}^{2}$ and ${\widetilde{R}}_{Y}^{2}$, which represent the proportions of the original variances explained by the unobserved confounders for the mediator and the outcome, respectively. The zero-contour line corresponds to ${\widetilde{R}}_{M}^{2}$ and ${\widetilde{R}}_{Y}^{2}$ values that yielded a zero-average causal mediation effect. For example, when ${\widetilde{R}}_{M}^{2}$=0.5 and ${\widetilde{R}}_{Y}^{2}$=0.3, the estimated mediation effect would be approximately zero. This means that the unobserved confounding ability would have to explain 50% of the original variance in the (latent) mediator variable and 30% of the original variance in the outcome variable for the estimate to be zero. This implies that the values of ${\widetilde{R}}_{M}^{2}$ and ${\widetilde{R}}_{Y}^{2}$. They must be relatively high to reverse the original conclusions.

## Discussion

This prospective birth cohort study showed that an IPI less than 12 months was independently associated with neurodevelopment in infants after adjusting for prenatal and postnatal confounding factors. There was a positive association between short IPI and ASQ failure in communication, fine motor, and personal-social domains. Furthermore, our findings suggest that maternal–fetal glucose metabolism disorder may mediate the association of short IPI with a failure communication domain. To the best of our knowledge, the present study provides the first evidence of possible glucose metabolism pathways by which a short IPI is associated with neurodevelopment in infants.

Our study found that IPIs < 3, 3–5, and 6–11 months were associated with a higher risk of neurodevelopmental delay in infants, using IPIs ≥ 24 months as a reference and that the risk values were essentially the same for the three groups. An IPI < 12 months has been associated with the highest risk of poor neurodevelopment in similar studies performed in California and Finland [19, 20], which is consistent with our conclusion. Our findings in infants suggest that a short IPI is associated with the risk of neurodevelopmental delay after controlling for a variety of confounders including sociodemographic characteristics, perinatal health status and lifestyle, birth outcomes, and postnatal factors. Our findings are consistent with previously reported associations between a short IPI and neurodevelopmental outcomes, such as ASD and severe mental illness [6, 29]. Specific potential confounders, such as folic acid supplementation, duration of breastfeeding, and postnatal factors have been reported to influence neurodevelopment in infants [30, 31].

The maternal–fetal glucose metabolism may be a possible biological factor in neurodevelopmental abnormalities associated with IPI. Studies have shown that maternal metabolic disorders increase signs of fetal B-cell hyperactivity [32]. Furthermore, insulin resistance is associated with higher levels of C-peptides and several neonatal complications such as fetal hyperinsulinemia [15]. Epidemiological evidence suggests that neonatal hypoglycemia is associated with an increased risk of neurodevelopmental delays, particularly cognitive dysfunction [33]. Based on our pre-cohort findings, maternal blood glucose, and cord blood C-peptide levels were associated with neurodevelopmental delays in infants [19, 20]. An experimental study in rats found that prolonged hypoglycemia, in which glucose supply is compromised, and compensatory changes that maintain cerebral oxidative metabolism are initiated in the brain, lead to neuronal damage when all amino acids, such as glutamate, are depleted and the rate of ATP production is no longer able to support the brain's minimum energy requirements [17]. Thus, a short IPI may affect fetal islet function and influence neurodevelopment in infants. In addition, we performed a mediation analysis that suggested that a role for maternal HOMA-IR and cord blood C-peptide levels in the association between short IPI and failing the communication domain.

Various biological pathways have been reported, besides maternal–fetal glucose metabolism. Biologically, the depletion of micronutrients after a short IPI can lead to the degradation of physiological functions and the transient nutritional depletion of nutrients, especially folic acid deficiency, which affects neurodevelopment [34]. Substantial evidence has linked poor maternal nutrition to brain development in infants [2]. Studies have shown that most pregnancies after a short IPI are unintended and that unintended pregnancies are associated with a higher risk of maternal stress during pregnancy [35]. Maternal psychological stress during pregnancy and folic acid deficiency may alter fetal DNA methylation, which may affect neural behavior [7, 36]. Another mechanism may involve maternal inflammation. It has been proposed that persistent maternal inflammation can improve fetal neurodevelopment when the pregnancy occurs at a relatively short IPI after delivery [20]. In some studies, maternal genital tract infections have been associated with an increased risk for ADHD, supporting a potential immune-related etiology [37]. At the same time, there is an increased risk of maternal complications in mothers who conceive after a shorter IPI as well as various changes in health-related behavior [5]. The advanced age of the parents can also contribute to poor neurodevelopment; however, the age of the parents was also considered in our analysis. Further research is required to better understand these mechanisms.

Notably, our results represent a composite measure of the risk of neurodevelopmental delay, including delays in communication and motor skills, problem-solving ability, and personal-social skills, rather than capturing specific neurodevelopmental outcomes. Our study found a positive correlation between short IPI and ASQ-3 failure in the communication, fine motor, and personal-social domains. We also found that a short IPI score was associated with a higher risk of neurodevelopmental delay in children. The communication domain of the ASQ-3 has been used as a proxy measure to determine which children are at increased risk of ASD diagnosis. Personal-social and fine motor domains are associated with ASD, attention-deficit/hyperactivity disorder (ADHD), academic performance, and the risk of brain damage [10, 38]. Neurodevelopmental delays in early childhood are often associated with tardive development of severe mental disorders [22]. Therefore, comprehensive measures for early neurodevelopment in children are of great interest.

Our study has several strengths. First, it was a population-based prospective cohort study with a relatively large sample size, making the results more stable. Second, we controlled for various confounders during pregnancy and infancy, including breastfeeding duration and folic acid intake, which directly affect neurological development. Third, we found that maternal–fetal glucose metabolism disorders partly mediated the association between short IPI and the risk of neurodevelopmental delay in infants. To the best of our knowledge, the present study provides the first evidence of the involvement of a possible glucose metabolism disorder pathway by which a shorter IPI is associated with neurodevelopment. Finally, our results also indicate there is an increased risk of childhood neurodevelopmental delay. Of note, our proposed formula represents a composite measure of early childhood risk of neurodevelopmental delay, including communication and motor skills, problem-solving ability, and personal and social skills, rather than focussing on a specific neurodevelopmental outcome.

A limitation of our study is that, as in any observational epidemiological study, it cannot point to a causal relationship between IPI and the risk of neurodevelopmental delay. Although we controlled for a series of maternal and infant characteristics, in the present study, nonetheless, different methodologies limit the interpretation of these findings, and unmeasured confounders that may influence neurological development were not possible, such as environmental factors (heavy metal and benzene exposure) and genetic factors (psychiatric or mental illness in family members) [39, 40]. Second, women who previously birthed a child with neurological defects often choose a longer IPI to reduce the risk of neurodevelopmental delay, leading to an underestimation of the effect of a short IPI. Third, it may cause some artificial bias because it is a parent/caregiver-completed screening questionnaire rather than a clinical diagnosis tool, although the ASQ is a general tool to assess infant neurodevelopment worldwide. Fourth, with delays in any 2 domains and delays in all 3 domains having smaller sample sizes and incidence rates of less than 5%, the relatively small sample size may have resulted in wider confidence intervals. Nonetheless, this study conducted sensitivity analyses using metrics from Poisson regression analyses and concluded that these associations were remain significant. Finally, infants’ neurodevelopment was evaluated at a single developmental stage, and further research is needed to estimate this association in childhood and adolescence.

## Conclusions

In this prospective birth cohort study, we provide new evidence that maternal–fetal glucose metabolism partly mediates the association of short IPI with neurodevelopmental. From a public health perspective, a combination of broader studies is needed to propose appropriate IPIs to reduce the risk of neurodevelopment. On the other hand, in clinical practice, for IPI < 12 months, maternal glucose metabolism needs to be closely monitored. Notably, the limited sample size in this study resulted in partially wider confidence intervals of effect size, more cohort studies with large sample are still required to validate them. Future intervention studies could target maternal–fetal glucose metabolism to reduce the risk of neurodevelopmental delay in infants.

### Supplementary Information

**Additional file 1: Table S1.** Association of covariates with infant neurodevelopment (*n* = 2599 ). **Table S2.** Association of covariates with maternal HOMA-IR and cord blood C-peptide [β (95% CI)]. **Table S3.** Characteristics of participants and non-Participants [Mean (SD) or n (%)]. **Table S4.** Associations of the IPI with infant neurodevelopment. **Table S5.** Adjusted associations of the IPI with infant neurodevelopment. **Fig. S1.** Flow diagram for the recruitment of mother-infant pairs in the prospective follow-up study. **Fig. S2.** Directed acyclic graph for the association between IPI and infant neurodevelopment. **Fig. S3.** IPI about maternal HOMA-IR and cord blood C-peptide. A) Box plot of IPI and maternal HOMA-IR. B) Box plot of IPI and cord blood C-peptide. C) Scatter plot of maternal HOMA-IR and cord blood C-peptide. **Fig. S4.** Cubic spline plots of IPI and neurodevelopment. The solid line indicates the nonlinear relationship between IPI and risk of neurodevelopmental delay, and the shaded area indicates the confidence interval. **Fig. S5.** IPI about maternal HOMA-IR and cord blood C-peptide. A) Box plot of IPI and maternal HOMA-IR. B) Box plot of IPI and cord blood C-peptide. **, *P* <0.05; ***, *P* <0.01; ns, *P* >0.05. **Fig. S6.** Sensitivity analysis with binary outcome and continuous mediator. The dashed line in the left plot represents the estimated mediation effect for *ρ* = 0. The gray areas represent the 95% confidence interval for the mediation effects at each value of *ρ*. The solid line represents the estimated average mediation effect at different values of ρ. The right plot contains contour lines that represent estimated average mediation effect corresponding to unobserved pretreatment confounders of various magnitudes. These magnitudes are measured by the coefficients of determination, ${\widetilde{\mathrm{R}}}_{\mathrm{M}}^{2}$ and ${\widetilde{\mathrm{R}}}_{\mathrm{Y}}^{2}$, each of which represents the proportion of original variance explained by the unobserved confounder for the mediator and the outcome, respectively.

## Data Availability

The original contributions presented in the study are included in the article/Supplementary material, further inquiries can be directed to the corresponding author.

## Abbreviations

ADE: Average direct effect
ADHD: Attention-deficit/hyperactivity disorder
AME: Average mediation effect
ASD: Autism spectrum disorder
ASQ-3: Ages and Stages Questionnaire Edition 3
BISQ: Brief Infant Sleep Questionnaire
BMI: Body mass index
CI: Confidence interval
DBP: Diastolic blood pressure
FPG: Fasting plasma glucose
HOMA-IR: Homeostasis model assessment of insulin resistance
IPI: Interpregnancy interval
IRR: Incident rate ratio
OGTT: 75-G oral-glucose-tolerance test
RMB: Chinese yuan
RR: Relative risk
SBP: Systolic blood pressure
SD: Standard deviation
USD: United States Dollar

## References

[1] Regan AK, Gissler M, Magnus MC, Håberg SE, Ball S, Malacova E (2019). Association between interpregnancy interval and adverse birth outcomes in women with a previous stillbirth: an international cohort study. Lancet.

[2] Gunnes N, Surén P, Bresnahan M, Hornig M, Lie KK, Lipkin WI (2013). Interpregnancy Interval and Risk of Autistic Disorder. Epidemiology (Cambridge, Mass)..

[3] Li H, Fan C, Mubarik S, Nabi G, Ping YX, Nawsherwan (2022). The trend in delayed childbearing and its potential consequences on pregnancy outcomes: a single center 9-years retrospective cohort study in Hubei, China. BMC Pregnancy Childb.

[4] Tatum M (2021). China's three-child policy. LANCET.

[5] Schieve LA, Tian LH, Drews-Botsch C, Windham GC, Newschaffer C, Daniels JL (2018). Autism spectrum disorder and birth spacing: Findings from the study to explore early development (SEED). Autism Res.

[6] Gunawardana L, Smith GD, Zammit S, Whitley E, Gunnell D, Lewis S (2011). Pre-conception inter-pregnancy interval and risk of schizophrenia. Brit J Psychiat.

[7] Conde-Agudelo A, Rosas-Bermudez A, Norton MH. Birth Spacing and Risk of Autism and Other Neurodevelopmental Disabilities: A Systematic Review. Pediatrics. 2016;137(5):e20153482. 10.1542/peds.2015-3482 27244802

[8] Duggan C, Irvine AD, O'B HJ, Kiely ME, Murray DM. ASQ-3 and BSID-III's concurrent validity and predictive ability of cognitive outcome at 5 years. Pediatr Res. 2023;94(4):1465–71. 10.1038/s41390-023-02528-y PMC10589087 36841883

[9] Yadama AP, Kelly RS, Lee-Sarwar K, Mirzakhani H, Chu SH, Kachroo P (2020). Allergic disease and low ASQ communication score in children. Brain Behav Immun.

[10] Grant R, Fléchelles O, Tressières B, Dialo M, Elenga N, Mediamolle N (2021). In utero Zika virus exposure and neurodevelopment at 24 months in toddlers normocephalic at birth: a cohort study. BMC Med.

[11] Hanley GE, Hutcheon JA, Kinniburgh BA, Lee L (2017). Interpregnancy Interval and Adverse Pregnancy Outcomes: An Analysis of Successive Pregnancies. Obstet Gynecol.

[12] Tinius RA, Blankenship MM, Furgal KE, Cade WT, Pearson KJ, Rowland NS (2020). Metabolic flexibility is impaired in women who are pregnant and overweight/obese and related to insulin resistance and inflammation. Metabolism.

[13] Wang P, Xie J, Jiao XC, Ma SS, Liu Y, Yin WJ (2021). Maternal Glycemia During Pregnancy and Early Offspring Development: A Prospective Birth Cohort Study. J Clin Endocr Metab.

[14] Sanguinetti E, Liistro T, Mainardi M, Pardini S, Salvadori PA, Vannucci A (2016). Maternal high-fat feeding leads to alterations of brain glucose metabolism in the offspring: positron emission tomography study in a porcine model. Diabetologia.

[15] Eid J, Kechichian T, Benavides E, Thibodeaux L, Salazar AE, Saade GR (2022). The Quantose Insulin Resistance Test for Maternal Insulin Resistance: A Pilot Study. Am J Perinat.

[16] Avatapalle HB, Banerjee I, Shah S, Pryce M, Nicholson J, Rigby L, et al. Abnormal Neurodevelopmental Outcomes are Common in Children with Transient Congenital Hyperinsulinism. Front Endocrinol. 2013;4:60. 10.3389/fendo.2013.00060 PMC3657691 23730298

[17] Rao R, Ennis K, Long JD, Ugurbil K, Gruetter R, Tkac I (2010). Neurochemical changes in the developing rat hippocampus during prolonged hypoglycemia. J Neurochem.

[18] Yin W, Yu L, Wu L, Zhang L, Li Q, Dai F, et al. Adequate 25(OH)D moderates the relationship between dietary inflammatory potential and cardiovascular health risk during the second trimester of pregnancy. Front Nutr. 2022;9:952652. 10.3389/fnut.2022.952652 PMC9372498 35967812

[19] Cheslack-Postava K, Liu K, Bearman PS (2011). Closely spaced pregnancies are associated with increased odds of autism in California sibling births. Pediatrics.

[20] Cheslack-Postava KP, Suominen AM, Jokiranta EM, Lehti VM, McKeague IWP, Sourander AM (2014). Increased Risk of Autism Spectrum Disorders at Short and Long Interpregnancy Intervals in Finland. J Am Acad Child Psy.

[21] Chen X, Chu C, Doebis C, von Baehr V, Hocher B (2021). Sex-Dependent Association of Vitamin D With Insulin Resistance in Humans. J Clin Endocr Metab.

[22] Girchenko P, Lahti-Pulkkinen M, Heinonen K, Reynolds RM, Laivuori H, Lipsanen J (2020). Persistently High Levels of Maternal Antenatal Inflammation Are Associated With and Mediate the Effect of Prenatal Environmental Adversities on Neurodevelopmental Delay in the Offspring. Biol Psychiatr (1969)..

[23] Anhui Statistical Yearbook 2022. http://tjj.ah.gov.cn/oldfiles/tjj/tjjweb/tjnj/2022/index.htm. Accessed 10 Aug 2023.

[24] Craig CL, Marshall AL, Sjöström M, Bauman AE, Booth ML, Ainsworth BE (2003). International physical activity questionnaire: 12-country reliability and validity. Med Sci Sport Exer.

[25] Tauman R, Levine A, Avni H, Nehama H, Greenfeld M, Sivan Y (2011). Coexistence of sleep and feeding disturbances in young children. Pediatrics (Evanston).

[26] Sadeh A (2004). A brief screening questionnaire for infant sleep problems: validation and findings for an Internet sample. Pediatrics.

[27] Zhang J, Yu KF (1998). What's the relative risk? A method of correcting the odds ratio in cohort studies of common outcomes. JAMA-J Am Med Assoc.

[28] Imai K, Keele L, Tingley D (2010). A general approach to causal mediation analysis. Psychol Methods.

[29] Zerbo O, Yoshida C, Gunderson EP, Dorward K, Croen LA (2015). Interpregnancy Interval and Risk of Autism Spectrum Disorders. Pediatrics (Evanston).

[30] McNulty H, Rollins M, Cassidy T, Caffrey A, Marshall B, Dornan J, et al. Effect of continued folic acid supplementation beyond the first trimester of pregnancy on cognitive performance in the child: a follow-up study from a randomized controlled trial (FASSTT Offspring Trial). BMC Med. 2019;17(1):196. 10.1186/s12916-019-1432-4 PMC6823954 31672132

[31] Boucher O, Julvez J, Guxens M, Arranz E, Ibarluzea J, Sánchez DMM (2017). Association between breastfeeding duration and cognitive development, autistic traits and ADHD symptoms: a multicenter study in Spain. Pediatr Res.

[32] Fallucca F, Maldonato A, Iavicoli M, Di Rollo G, Di Biase N, Napoli A (1989). Influence of maternal metabolic control and insulin antibodies on neonatal complications and B cell function in infants of diabetic mothers. Diabetes Res Clin Pr.

[33] Wickström R, Skiöld B, Petersson G, Stephansson O, Altman M (2018). Moderate neonatal hypoglycemia and adverse neurological development at 2–6 years of age. Eur J Epidemiol.

[34] Pereira G, Francis RW, Gissler M, Hansen SN, Kodesh A, Leonard H (2021). Optimal interpregnancy interval in autism spectrum disorder: A multi-national study of a modifiable risk factor. Autism Res.

[35] McCrory C, McNally S (2013). The Effect of Pregnancy Intention on Maternal Prenatal Behaviours and Parent and Child Health: Results of an Irish Cohort Study. Paediatr Perinat Ep.

[36] Sammallahti S, Cortes Hidalgo AP, Tuominen S, Malmberg A, Mulder RH, Brunst KJ (2021). Maternal anxiety during pregnancy and newborn epigenome-wide DNA methylation. Mol Psychiatr.

[37] Werenberg Dreier J, Nybo Andersen AM, Hvolby A, Garne E, Kragh Andersen P, Berg BG (2016). Fever and infections in pregnancy and risk of attention deficit/hyperactivity disorder in the offspring. J Child Psychol Psyc.

[38] Bos AF, Van Braeckel KNJA, Hitzert MM, Tanis JC, Roze E (2013). Development of fine motor skills in preterm infants. Dev Med Child Neurol.

[39] Webb E, Moon J, Dyrszka L, Rodriguez B, Cox C, Patisaul H (2018). Neurodevelopmental and neurological effects of chemicals associated with unconventional oil and natural gas operations and their potential effects on infants and children. Rev Environ Health.

[40] Grotzinger AD, Mallard TT, Akingbuwa WA, Ip HF, Adams MJ, Lewis CM (2022). Genetic architecture of 11 major psychiatric disorders at biobehavioral, functional genomic and molecular genetic levels of analysis. Nat Genet.

