# Supplementary material for: Interpregnancy interval and early infant neurodevelopment: the role of maternal–fetal glucose metabolism
Source: BMC Med. 2024 Jan 2;22:2. doi: 10.1186/s12916-023-03191-0 (PMC10762827; doi:10.1186/s12916-023-03191-0)
Supplement: Supplementary file 1 — Additional file 1: Table S1. Association of covariates with infant neurodevelopment (n = 2599 ). Table S2. Association of covariates with maternal HOMA-IR and cord blood C-peptide [β (95% CI)]. Table S3. Characteristics of participants and non-Participants [Mean (SD) or n (%)]. Table S4. Associations of the IPI with infant neurodevelopment. Table S5. Adjusted associations of the IPI with infant neurodevelopment. Fig. S1. Flow diagram for the recruitment of mother-infant pairs in the prospective follow-up study. Fig. S2. Directed acyclic graph for the association between IPI and infant neurodevelopment. Fig. S3. IPI about maternal HOMA-IR and cord blood C-peptide. A) Box plot of IPI and maternal HOMA-IR. B) Box plot of IPI and cord blood C-peptide. C) Scatter plot of maternal HOMA-IR and cord blood C-peptide. Fig. S4. Cubic spline plots of IPI and neurodevelopment. The solid line indicates the nonlinear relationship between IPI and risk of neurodevelopmental delay, and the shaded area indicates the confidence interval. Fig. S5. IPI about maternal HOMA-IR and cord blood C-peptide. A) Box plot of IPI and maternal HOMA-IR. B) Box plot of IPI and cord blood C-peptide. **, P <0.05; ***, P <0.01; ns, P >0.05. Fig. S6. Sensitivity analysis with binary outcome and continuous mediator. The dashed line in the left plot represents the estimated mediation effect for ρ = 0. The gray areas represent the 95% confidence interval for the mediation effects at each value of ρ. The solid line represents the estimated average mediation effect at different values of ρ. The right plot contains contour lines that represent estimated average mediation effect corresponding to unobserved pretreatment confounders of various magnitudes. These magnitudes are measured by the coefficients of determination, [file 12916_2023_3191_MOESM1_ESM.docx]

**Interpregnancy interval and early infant neurodevelopment: the role of maternal-fetal glucose metabolism**

Ruirui Ma^1,2,3,4,†^, Peng Wang^1,2,3,4,†^, Qiaolan Yang^5,†^, Yuanyuan Zhu^1,2,4^, Lei Zhang^1,2,4^, Yuhong Wang^1,2,4^, Lijun Sun^1^, Wenxiang Li^1^, Jinfang Ge^6,*^, Peng Zhu^1,2,3,4,*^

**Author affiliations:**

^1^ Department of Maternal, Child and Adolescent Health, School of Public Health, Anhui Medical University, Hefei, China

^2^ MOE Key Laboratory of Population Health Across Life Cycle, Hefei, China

^3^ NHC Key Laboratory of Study on Abnormal Gametes and Reproductive Tract, Anhui Medical University, Hefei, China

^4^ Anhui Provincial Key Laboratory of Population Health and Aristogenics, Anhui Medical University, Hefei, China

^5^ Department of Urology, The First Affiliated Hospital of Anhui Medical University, Hefei, China

^6^ School of Pharmacy, Anhui Medical University, Hefei, China

**Correspondence:**

Jinfang Ge, PhD, School of Pharmacy, Anhui Medical University, 81 Meishan Road, Hefei, Anhui 230032, China. E-mail: gejinfang@ahmu.edu.cn. Phone: 0551 65172131;

Peng Zhu, Department of Maternal, Child and Adolescent Health, School of Public Health, Anhui Medical University, 81 Meishan Road, Hefei, Anhui 230032, China. Email: pengzhu@ahmu.edu.cn. Phone: 0551 65161169.

**Table S1.** Association of covariates with infant neurodevelopment (n = 2599 ).

|  | Delay in one ASQ domain, [*RR* (95% *CI*)] | | | | |
| --- | --- | --- | --- | --- | --- |
|  | Communication | Gross motor | Fine motor | Problem solving | Personal social |
| **Sociodemographic characteristics** |  |  |  |  |  |
| Maternal age, years | 0.996(0.926,1.070) | 1.008(0.934,1.088) | 1.022(0.950,1.098) | 1.067(0.968,1.176) | 0.979(0.918,1.044) |
| Maternal education <12 years | 0.975(0.554,1.717) | 0.928(0.511,1.686) | 0.975(0.554,1.717) | 0.495(0.234,1.048) | 1.157(0.690,1.939) |
| Income <4000 RMB/month | 0.649(0.329,1.281) | 0.506(0.233,1.098) | 1.103(0.602,2.021) | 0.780(0.329,1.847) | 1.104(0.640,1.906) |
| **Perinatal health status and lifestyle** |  |  |  |  |  |
| Parity ≥3 | 1.320(0.754,2.311) | 1.121(0.620,2.026) | 0.953(0.545,1.669) | 1.386(0.659,2.912) | 1.325(0.800,2.195) |
| Prepregnancy overweight/obesity | 1.463(0.787,2.718) | 0.980(0.478,2.007) | 1.614(0.878,2.967) | 1.614(0.724,3.596) | 0.788(0.413,1.502) |
| SBP, mmHg | 0.994(0.965,1.023) | 1.002(0.972,1.033) | 1.005(0.977,1.034) | 1.007(0.969,1.045) | 0.980(0.954,1.006) |
| DBP, mmHg | 1.014(0.977,1.054) | 0.998(0.959,1.040) | 1.000(0.962,1.039) | 1.017(0.967,1.069) | 0.983(0.962,1.004) |
| Depression during pregnancy | **1.126(1.050,1.207)** | 0.973(0.899,1.054) | **1.105(1.031,1.185)** | **1.104(1.007,1.209)** | **1.070(1.004,1.141)** |
| Gestational diabetes mellitus | 1.233(0.646,2.355) | 0.955(0.604,1.510) | 0.733(0.460,1.170) | 1.169(0.493,2.775) | 1.004(0.545,1.852) |
| Anemia during pregnancy | 0.612(0.330,1.137) | 0.974(0.529,1.794) | 0.941(0.528,1.676) | 0.548(0.240,1.250) | 1.193(0.718,1.980) |
| FPG, mmol/L | 1.295(0.725,2.316) | 0.597(0.300,1.189) | 0.739(0.505,1.081) | 1.341(0.627,2.871) | 0.789(0.449,1.387) |
| Insulin, IU/L | **1.058(1.005,1.114)** | 0.999(0.920,1.085) | 0.970(0.890,1.058) | 1.024(0.938,1.118) | 0.967(0.893,1.046) |
| Vitamin D supplement | 1.508(0.854,2.662) | 0.692(0.380,1.261) | 1.177(0.672,2.060) | 0.807(0.384,1.697) | 0.906(0.548,1.499) |
| Folic acid supplement | 0.876(0.496,1.546) | 1.098(0.606,1.989) | 1.034(0.589,1.815) | 1.879(0.887,3.977) | 0.984(0.592,1.635) |
| Physical activity <3 days/week | 0.892(0.507,1.570) | 1.236(0.684,2.235) | 0.892(0.507,1.570) | 0.860(0.407,1.821) | 0.751(0.448,1.259) |
| **Birth outcomes** |  |  |  |  |  |
| Cesarean section | 1.250(0.690,2.265) | 1.478(0.799,2.734) | 1.580(0.892,2.800) | 1.570(0.729,3.382) | 0.626(0.351,1.114) |
| Male | 0.535(0.286,1.003) | 1.517(0.819,2.810) | 0.708(0.394,1.274) | 1.096(0.509,2.359) | 0.646(0.376,1.107) |
| Gestational week at birth, week | 0.943(0.754,1.180) | 0.899(0.721,1.121) | 0.973(0.777,1.218) | 0.972(0.812,1.163) | 0.915(0.755,1.110) |
| Birth weight, g | 1.000(0.999,1.000) | 1.000(0.999,1.000) | 1.000(0.999,1.001) | 1.000(0.999,1.000) | 1.000(0.999,1.000) |
| **Postnatal factors** |  |  |  |  |  |
| Postpartum depression | 1.055(0.981,1.134) | 0.946(0.858,1.043) | 1.051(0.974,1.134) | 1.031(0.925,1.149) | 1.004(0.931,1.082) |
| Exclusive breastfeeding >6 months | **0.468(0.256,0.854)** | 1.139(0.523,2.483) | 0.701(0.366,1.340) | 0.518(0.232,1.156) | 1.057(0.553,2.019) |
| Feeding difficulties | **3.612(1.795,7.267)** | 1.756(0.718,4.298) | **3.244(1.558,6.754)** | **3.570(1.304,9.772)** | **2.232(1.090,4.570)** |
| Poor sleeping in infants | 1.800(0.875,3.703) | 0.626(0.212,1.851) | 1.118(0.492,2.538) | 1.115(0.355,3.505) | 0.916(0.410,2.045) |
| Fever in infants≥38.5°C | 1.314(0.453,3.813) | 1.537(0.438,5.392) | 0.726(0.237,2.227) | 0.568(0.093,3.460) | 0.476(0.136,1.671) |
| Parents as primary caregivers | 1.084(0.521,2.252) | 0.733(0.324,1.659) | 1.093(0.509,2.347) | 1.479(0.477,4.693) | 1.626(0.817,3.236) |

**Table S2.** Association of covariates with maternal HOMA-IR and cord blood C-peptide [*β* (95% *CI*)].

|  | Maternal HOMA-IR | Cord blood C-peptide, nmol/L |
| --- | --- | --- |
| **Sociodemographic characteristics** |  |  |
| Maternal age, years | -0.005(-0.014,0.003) | **-0.005(-0.008,-0.002)** |
| Maternal education <12 years | **-0.111(-0.176,-0.045)** | 0.004(-0.018,0.027) |
| Income <4000 RMB/month | -0.016(-0.086,0.053) | -0.009(-0.034,0.016) |
| **Perinatal health status and lifestyle** |  |  |
| Parity ≥3 | -0.014(-0.080,0.052) | -0.010(-0.033,0.013) |
| Prepregnancy BMI | **0.116(0.106,0.126)** | -0.002(-0.005,0.002) |
| SBP, mmHg | **0.023(0.019,0.026)** | 0.000(-0.001,0.001) |
| DBP, mmHg | **0.028(0.024,0.032)** | 0.000(-0.002,0.001) |
| Depression during pregnancy | -0.003(-0.011,0.005) | 0.002(-0.001,0.005) |
| Gestational diabetes mellitus | **0.830(0.753,0.906)** | **0.056(0.028,0.084)** |
| Anemia during pregnancy | **-0.280(-0.347,-0.214)** | **-0.025(-0.048,-0.002)** |
| Vitamin D supplement | 0.045(-0.022,0.111) | **-0.061(-0.083,-0.038)** |
| Folic acid supplement | **0.084(0.018,0.151)** | 0.019(-0.004,0.042) |
| Physical activity <3 days/week | -0.062(-0.128,0.003) | **-0.038(-0.061,-0.015)** |

**Table S3.** Characteristics of participants and non-Participants [Mean (SD) or n (%)].

|  | Participants (n=2599) | Non-Participants (n=2529) | *P* value |
| --- | --- | --- | --- |
| IPI <12month | 362(13.9%) | 355(14.0%) | 0.911 |
| Maternal age, years | 30.90(3.98) | 30.78(4.02) | 0.282 |
| Maternal education <12 years | 1174(45.9%) | 1195(47.3%) | 0.326 |
| Income <4000 RMB/month | 1836(71.7%) | 1736(68.6%) | **0.016** |
| Prepregnancy overweight/obesity | 570(21.9%) | 559(22.1%) | 0.882 |
| SBP, mmHg | 110.0(9.9) | 110.6(10.2) | 0.060 |
| DBP, mmHg | 68.8(7.3) | 69.1(7.6) | 0.235 |
| Gestational diabetes | 542(20.9%) | 502(19.9%) | 0.361 |
| Depressive symptoms | 322(12.4%) | 376(14.9%) | 0.098 |
| FPG, mmol/L | 4.58(0.5) | 4.52(0.5) | 0.202 |
| Insulin, IU/L | 8.0(5.1) | 7.7(3.3) | 0.123 |
| Folic acid supplement | 1082(42.3%) | 1070(42.3%) | 0.984 |
| Vitamin D supplement | 1404(54.9%) | 1365(54.0%) | 0.523 |
| Parity ≥3 | 1158(44.6%) | 1157(45.7%) | 0.390 |

**Table S4.** Associations of the IPI with infant neurodevelopment.

| ASQ-3 at 12 months | Interpregnancy interval, months (n=2599) | | | | | |
| --- | --- | --- | --- | --- | --- | --- |
|  | <3 (n=60) | 3-5 (n=140) | 6-11 (n=162) | 12-17 (n=301) | 18-23  (n=333) | ≥24  (n=1603) |
| Failure to Communication domain | 6(10.0%) | 14(10.0%) | 12(7.4%) | 13(4.3%) | 17(5.1%) | 74(4.6%) |
| Failure to Gross motor domain | 3(5.0%) | 7(5.0%) | 7(4.3%) | 15(5.0%) | 14(4.2%) | 73(4.6%) |
| Failure to Fine motor domain | 7(11.7%) | 12(8.6%) | 10(6.2%) | 15(5.0%) | 17(5.1%) | 74(4.6%) |
| Failure to Problem solving domain | 0(0.0%) | 6(4.3%) | 6(3.7%) | 12(4.0%) | 14(4.2%) | 41(2.6%) |
| Failure to Personal social domain | 7(11.7%) | 16(11.4%) | 13(8.0%) | 17(5.6%) | 19(5.7%) | 102(6.4%) |

**Table S5.** Adjusted associations of the IPI with infant neurodevelopment.

| ASQ at 12 months | Interpregnancy interval, months [*RR* (95% *CI*)] | | | | | |
| --- | --- | --- | --- | --- | --- | --- |
|  | <3 | 3-5 | 6-11 | 12-17 | 18-23 | ≥24 |
| **Delay in one ASQ domain** |  |  |  |  |  |  |
| Communication | **2.39(1.00,5.76)** | **2.21(1.18,4.14)** | **2.60(1.50,4.52)** | 1.07(0.58,1.96) | 1.22(0.71,2.12) | 1 |
| Gross motor | 1.21(0.37,3.97) | 1.25(0.56,2.79) | 1.16(0.55,2.46) | 1.15(0.65,2.03) | 1.01(0.56,1.82) | 1 |
| Fine motor | **2.97(1.30,6.79)** | **2.12(1.11,4.05)** | **2.36(1.33,4.18)** | 1.23(0.69,2.18) | 1.17(0.67,2.04) | 1 |
| Problem solving | **-** | 1.63(0.63,4.25) | 1.89(0.86,4.31) | 1.65(0.85,3.20) | 1.87(0.99,3.49) | 1 |
| Personal social | 2.04(0.99,4.61) | **1.87(1.05,3.33)** | **1.96(1.16,3.31)** | 0.90(0.52,1.55) | 0.96(0.58,1.60) | 1 |
| Delay in communication, fine motor, and personal social domain |  |  |  |  |  |  |
| Delay in any 1 domain | **2.11(1.12,3.97)** | **1.59(1.00,2.55)** | **1.59(1.03,2.45)** | 1.23(0.85,1.78) | 1.05(0.73,1.52) | 1 |
| Delay in any 2 domains | **3.40(1.15,9.99)** | **3.65(1.73,7.61)** | **3.65(1.83,7.30)** | 1.35(0.80,1.47) | 1.07(0.47,2.46) | 1 |
| Delay in all 3 domains | **5.79(1.61,20.85)** | **4.90(1.83,13.11)** | **8.32(3.75,18.47)** | 1.39(0.51,2.98) | 2.07(0.79,5.45) | 1 |
| **Number of delay across 3 domains** | **2.20(0.93,5.21)** | **2.38(1.34,4.24)** | **2.55(1.51,4.30)** | 1.16(0.47,1.57) | 1.04(0.61,1.78) |  |


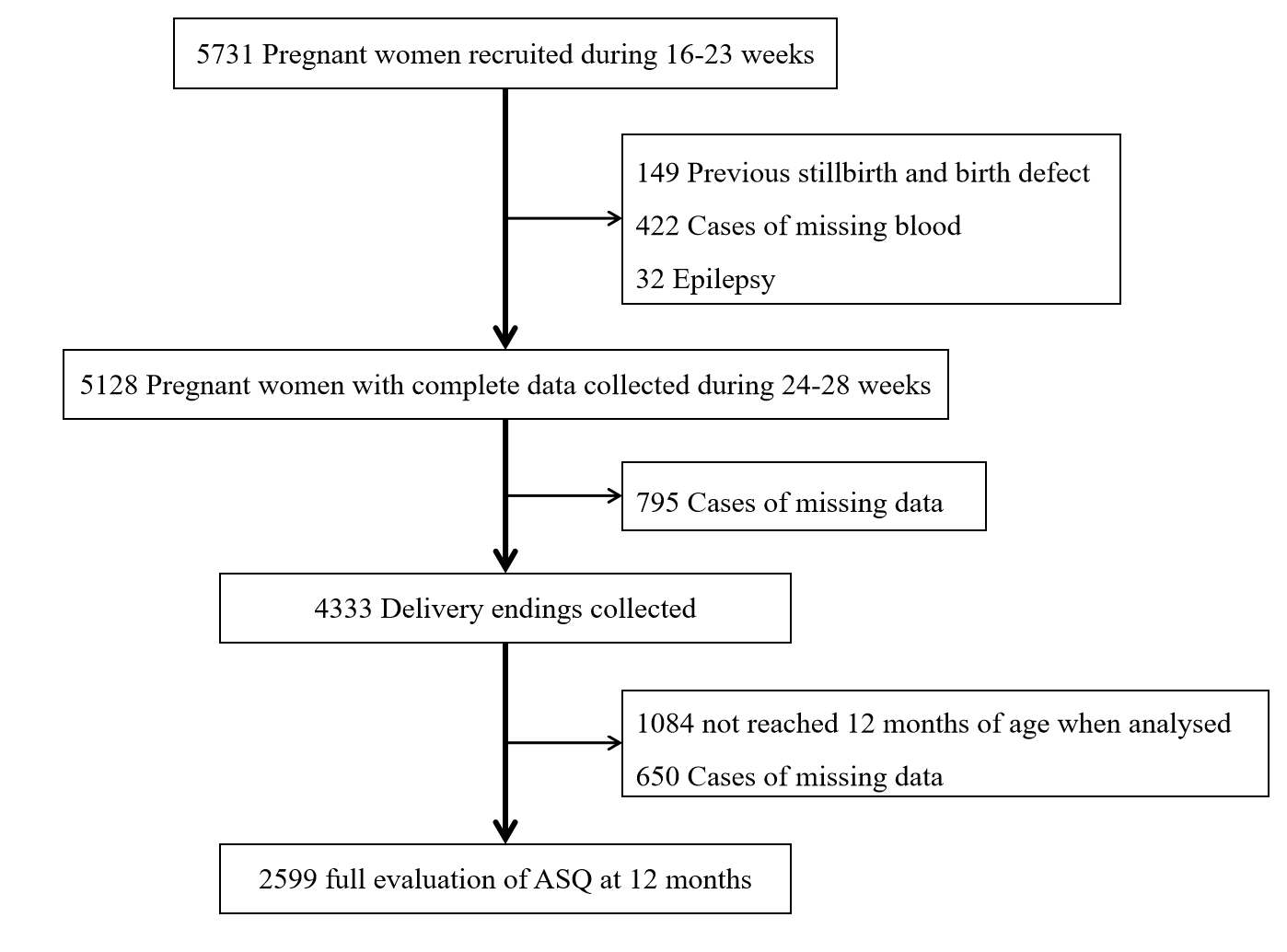


**Fig. S1.** Flow diagram for the recruitment of mother-infant pairs in the prospective follow-up study.


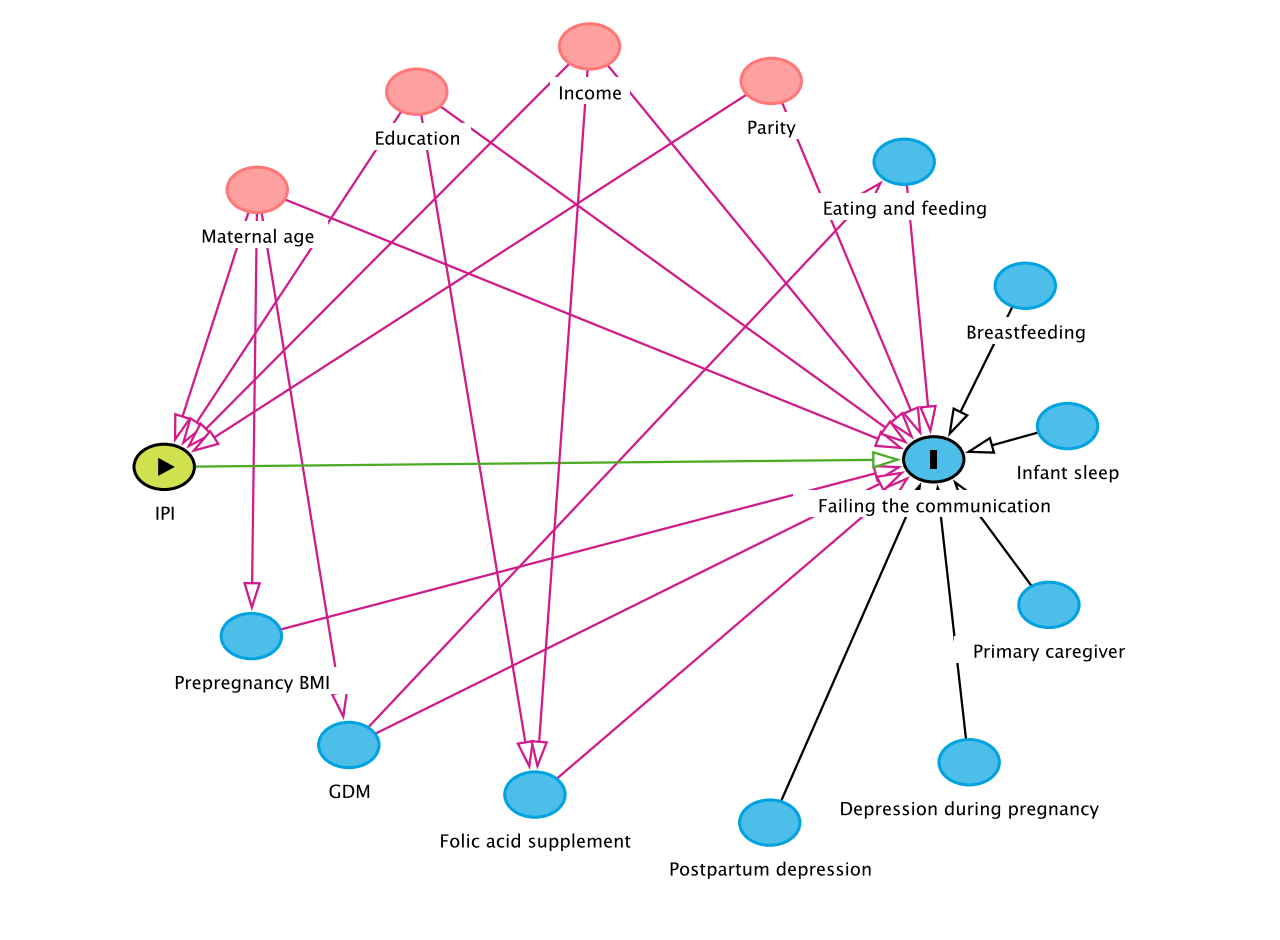


**Fig. S2.** Directed acyclic graph for the association between IPI and infant neurodevelopment.


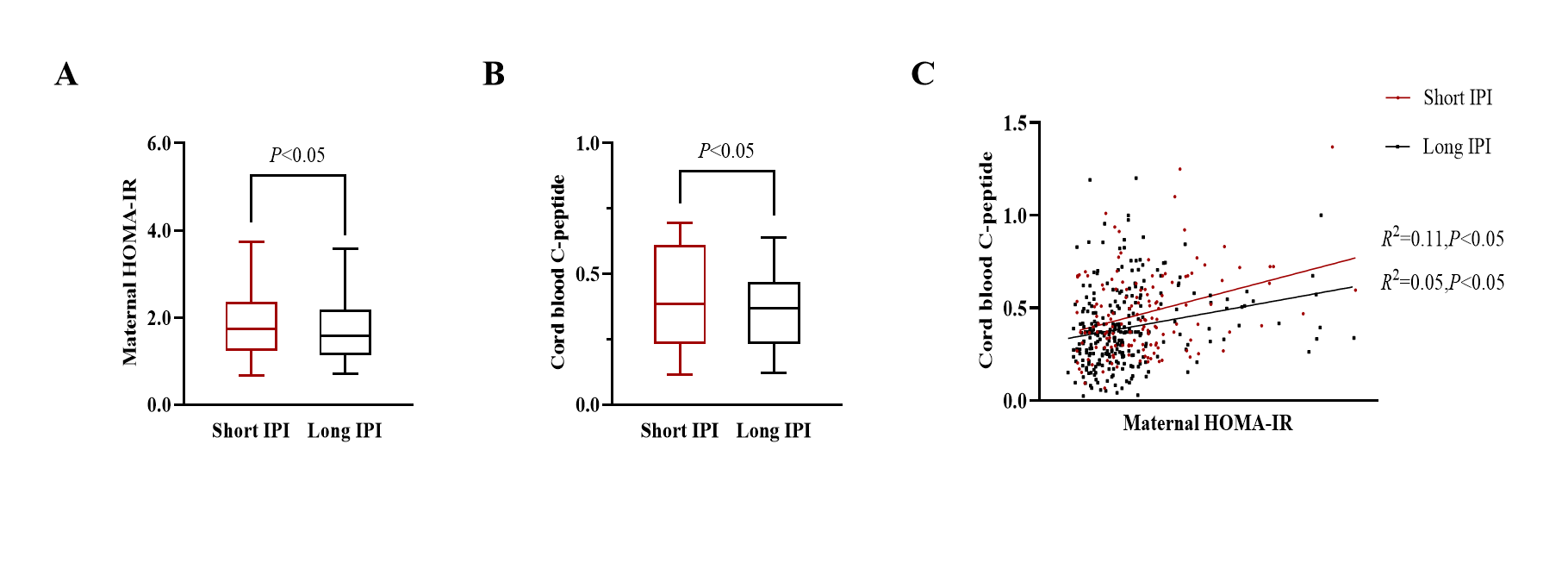


**Fig. S3.** IPI about maternal HOMA-IR and cord blood C-peptide. A) Box plot of IPI and maternal HOMA-IR. B) Box plot of IPI and cord blood C-peptide. C) Scatter plot of maternal HOMA-IR and cord blood C-peptide.


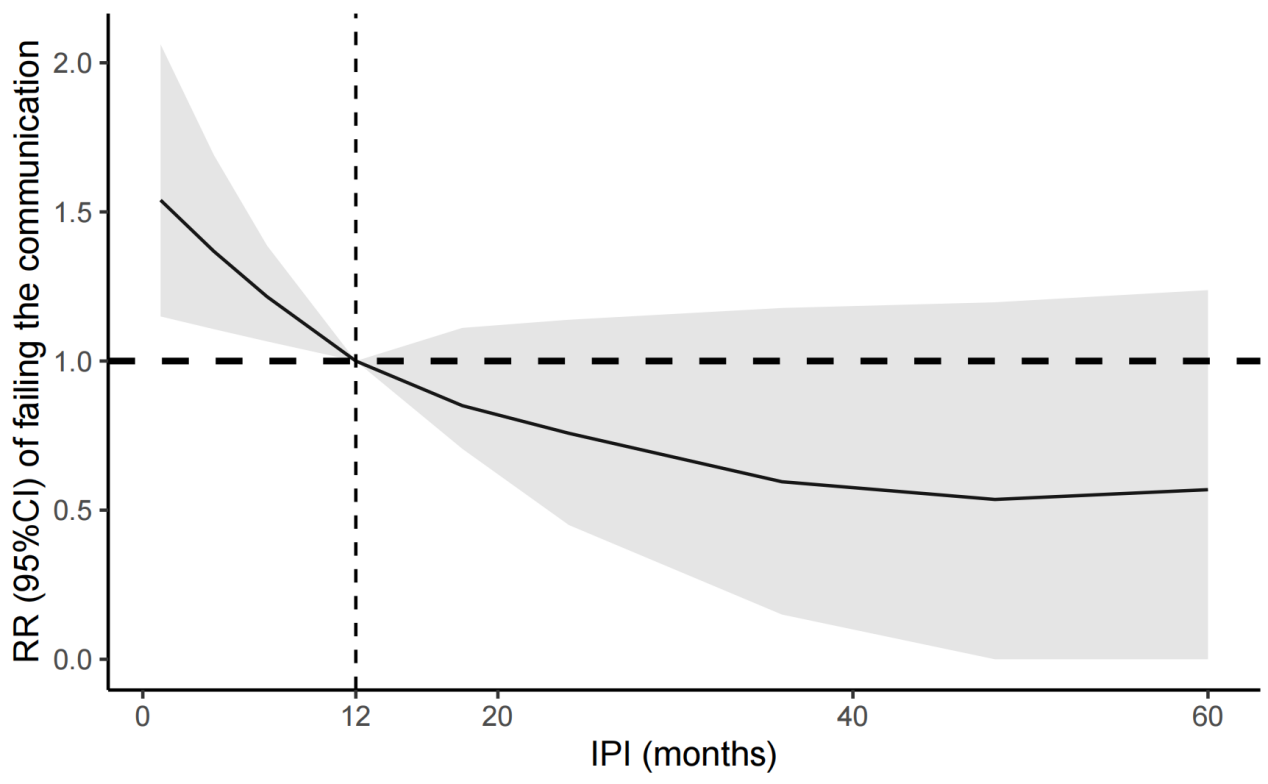


**Fig. S4.** Cubic spline plots of IPI and neurodevelopment. The solid line indicates the nonlinear relationship between IPI and risk of neurodevelopmental delay, and the shaded area indicates the confidence interval. There is a node when IPI = 12 months, IPI <12 months was associated with risk of neurodevelopmental delay, and ≥12 months was not statistically associated with risk of neurodevelopmental delay.


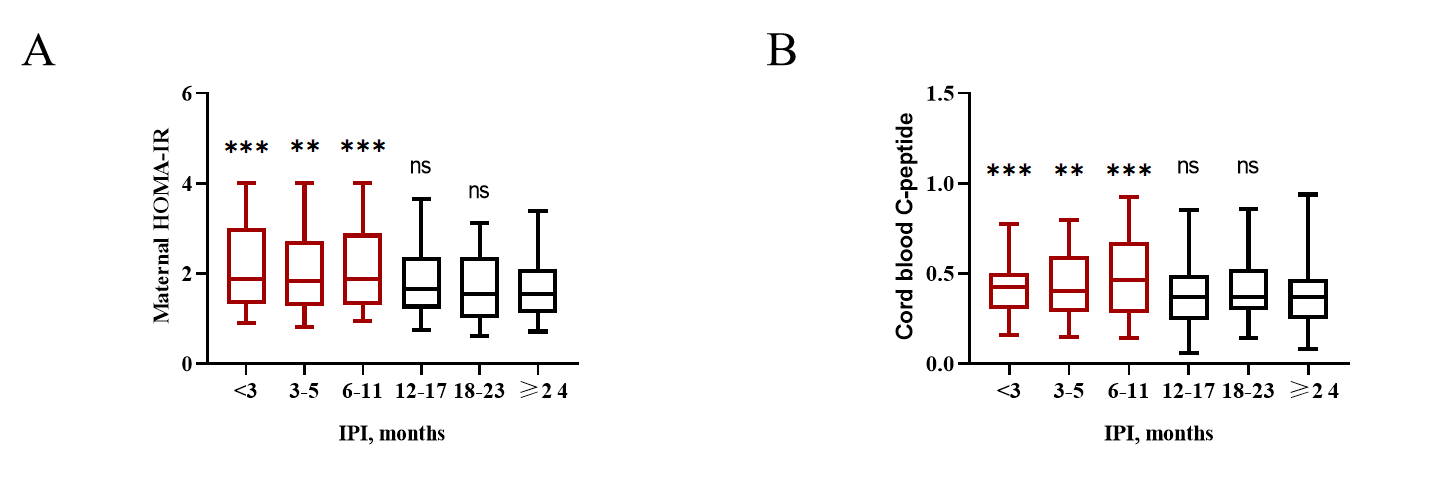


**Fig. S5.** IPI about maternal HOMA-IR and cord blood C-peptide. A) Box plot of IPI and maternal HOMA-IR. B) Box plot of IPI and cord blood C-peptide.

**, P <0.05; ***, P <0.01; ns, P >0.05.


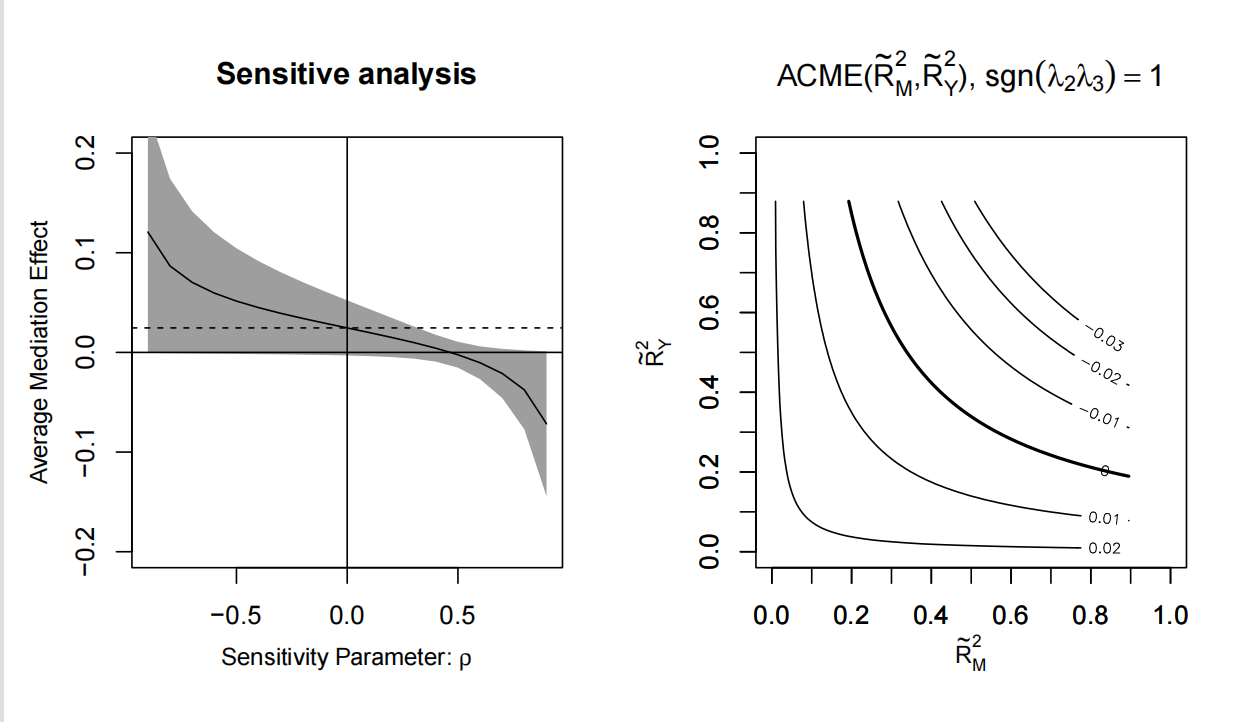
**Fig. S6.** Sensitivity analysis with binary outcome and continuous mediator. The dashed line in the left plot represents the estimated mediation effect for ρ = 0. The gray areas represent the 95% confidence interval for the mediation effects at each value of ρ. The solid line represents the estimated average mediation effect at different values of ρ. The right plot contains contour lines that represent estimated average mediation effect corresponding to unobserved pretreatment confounders of various magnitudes. These magnitudes are measured by the coefficients of determination, $\tilde{R}_{M}^{2}$ and $\tilde{R}_{Y}^{2}$, each of which represents the proportion of original variance explained by the unobserved confounder for the mediator and the outcome, respectively.
